# Around the Hospitals

**Published:** 1896-06-13

**Authors:** 


### Around the Hospitals.

**The Northampton Infirmary.**—The report of the past year at this infirmary is very satisfactory. The adverse balance of the previous year has been wiped out and the accounts show a balance in hand. The new buildings will be a great improvement, but will entail increased expenditure, so that it is to be hoped the receipts will show no falling off. A new system has been adopted in the method of electing the officers of the institution which promises to effect a distinct improvement. The number of in-patients treated amounted to 1,862, and the out-patients to 9,148. The income equalled £8,241, and the expenditure £7,302.

